# Effects of Bronchoalveolar Lavage with Ambroxol Hydrochloride on Treating Pulmonary Infection in Patients with Cerebral Infarction and on Serum Proinflammatory Cytokines, MDA and SOD

**DOI:** 10.1155/2020/7984565

**Published:** 2020-10-09

**Authors:** Fanhua Meng, Jing Cheng, Peng Sang, Jianhui Wang

**Affiliations:** ^1^Stroke Unit, The Affiliated Hospital of Beihua University, Jilin 132011, China; ^2^Respiratory Department, The Affiliated Hospital of Beihua University, Jilin 132011, China; ^3^Department of Rehabilitation Medicine, The Affiliated Hospital of Beihua University, Jilin 132011, China

## Abstract

**Objective:**

This paper was aimed at investigating the effects of bronchoalveolar lavage (BAL) with ambroxol hydrochloride (AH) on treating pulmonary infection and on serum proinflammatory cytokines and oxidative stress responses in patients with cerebral infarction (CI).

**Methods:**

One hundred and two patients with cerebral infarction complicated with pulmonary infection (CIPI) who were treated in our hospital were enrolled as research objects, divided into an observation group (52 cases; AH combined with BAL) and a control group (50 cases; single AH) based on therapeutic schemes. They were compared in terms of the therapeutic effect and pre- and posttreatment serum inflammatory cytokines, pulmonary function, and serum indices of oxidative stress. Their adverse reactions during treatment were also recorded and compared.

**Results:**

The therapeutic effect in the observation group was remarkably better than that in the control group (*P* < 0.05). After treatment, the serum inflammatory cytokines, pulmonary function, and serum indices of oxidative stress were remarkably improved in the two groups (*P* < 0.05), but the improvement was remarkably better in the observation group (*P* < 0.05). The differences were not significant in intratreatment adverse reactions between the two groups (*P* > 0.05).

**Conclusion:**

For CIPI patients, BAL with AH has a better therapeutic effect and higher safety and can control the patients' systemic inflammatory responses and oxidative stress responses, so it is worthy of further promotion in clinical practice.

## 1. Introduction

As a disease with a high incidence among the elderly, cerebral infarction (CI) is affected by many factors, with its clinical symptoms mostly accompanied by dysphagia and pharyngeal secretions that cannot be excluded [1, 2]. Many CI patients have low immunity, prone to be complicated with pulmonary infection [3]. Easy to cause further damages to the body, the complicated diseases affect the therapeutic effect on and the rehabilitation speed of the patients [4]. Moreover, due to the abuse of antibiotics and the emergence of drug-resistant strains, it is more difficult to treat CI complicated with pulmonary infection (CIPI) [5]. Therefore, it is of great clinical significance to seek effective therapeutic methods for CIPI patients.

At present, anti-infection treatment is mainly used for pulmonary infection, and the airway of patients should be kept unobstructed in order to improve pulmonary function [6]. CIPI patients experience expectoration disorders after pulmonary infection, and the viscous sputum in the lung is not easy to cough out, which easily results in pulmonary retention and even death in serious cases. Therefore, the rapid and effective control of pulmonary infection is crucial to treat CI patients [7]. Ambroxol hydrochloride (AH) is an expectorant, which is effective in dissolving viscous phlegm and lubricating the respiratory tract [8]. Having been widely used in the clinical treatment of pulmonary infection at present, bronchoalveolar lavage (BAL) is a therapeutic method to improve respiratory function and infection control via directly infusing drugs into diseased regions of pulmonary segments through a bronchoscope [9]. According to a previous study, AH combined with BAL has a better curative effect on patients with severe pneumonia and can improve their pulmonary function [10].

For seeking active and effective therapeutic methods for CIPI patients, we have explored the therapeutic effect of AH combined with BAL on the patients and obtained positive clinical results.

## 2. Materials and Methods

### 2.1. Clinical Data

A prospective analysis was made on 102 CIPI patients admitted to our hospital from February 2016 to October 2018, with an average age of 55.21 ± 2.62 years. Fifty cases in the control group received single AH, while 52 cases in the observation group received AH combined with BAL. All treatments were performed on the basis of conventional anti-infection treatment. Inclusion criteria include patients who were confirmed with CI by head CT or MRI and confirmed with pulmonary infection based on clinical manifestations, signs, laboratory examinations, and chest X-rays. Exclusion criteria include patients who were allergic to AH; patients who had used glucocorticoids for a long time; patients with severe hepatic and renal insufficiency; patients complicated with other malignant tumors; and patients who did not cooperate in treatment. All patients have consented to participation in the experiment and signed the informed consent. The Hospital Ethics Committee has agreed with this experiment.

### 2.2. Therapeutic Methods

All patients received conventional oxygen inhalation and anti-infection treatment. On this basis, those in the control group were intravenously dripped with 30 mg of AH (Sinopharm Group Guorui Pharmaceutical Co., Ltd., SFDA Approval Number: H20143385) twice per day. On the basis of the control group, those in the observation group were given BAL by an electronic bronchoscope once/day. Specific steps were as follows: before the operation, 2% lidocaine was atomized and inhaled to perform local anesthesia on the throat. After the flexible bronchofiberscope was connected with a negative pressure aspirator, high-concentration oxygen was inhaled for approximately 5 min under ECG monitoring. After blood oxygen saturation was ≥95%, the electronic bronchoscope was inserted through the nose, mouth, or artificial airway, with secretions in the airway sucked. Then, 0.9% sodium chloride solution (100 mL) + ambroxol hydrochloride injection (90 mg) was prepared for BAL, 10-20 mL once. During the operation, the negative pressure was controlled at ≤100 mmHg (1 mmHg = 0.133 kPa), and the actions should be gentle and rapid. Additionally, parameters of the ECG monitoring should be closely observed. The operation should be immediately stopped with the bronchoscope exited and oxygen inhaled, if the blood oxygen saturation was <85%. The operation should be continued if the blood oxygen saturation was ≥95%. Repeated lavage was carried out on each diseased pulmonary segment until the bronchoalveolar lavage fluid was clean. The lavage was conducted for ≤3 times, and the lobes on each side were lavaged with approximately 50 mL of the fluid. The patients in both groups were consecutively treated for 1 week.

### 2.3. Outcome Measures

(1) After treatment, the therapeutic effects on the patients were evaluated, which were divided into cured (the patients' clinical signs, laboratory examinations, and pathogen examinations showed recovery), markedly effective (the clinical symptoms were obviously relieved, but laboratory or pathogen examinations showed incomplete recovery), effective (the clinical symptoms were relieved but the relief was not very obvious), and ineffective (the clinical symptoms were not obviously relieved). Total effective rate = (number of cured cases + number of markedly effective cases)/total number of cases x 100%. (2) The patients' pulmonary function before and after treatment was assessed and compared between the two groups. MasterScreen PFT System was used to evaluate pulmonary function indices, which included forced vital capacity (FVC), forced expiratory volume in 1 s (FEV1), and FEV1 to FVC (FEV1/FVC). (3) ELISA was used to detect and compare levels of TNF-*α*, IL-8, and IL-6 before and after treatment between the two groups. (4) ELISA was also used to detect contents of serum indices of oxidative stress malondialdehyde (MDA) and superoxide dismutase (SOD) before and after treatment between the two groups. (5) The adverse reactions of the patients during treatment were recorded and compared, including increased heart rate, small amount of hemoptysis, decreased blood oxygen, and decreased heart rate

### 2.4. Statistical Methods

In this study, SPSS19.0 was applied to analyze the experimental data statistically. We use the chi-squared test to count data. Measurement data were expressed by mean ± standard deviation, and *t*-test was applied for the comparison between two groups and paired *t*-test for the comparison between before and after treatment. GraphPad Prism 6 was used for plotting figures in this experiment. *P* value < 0.05 was recognized as statistically significant.

## 3. Results

### 3.1. General Information

The differences were not significant in gender, age, body mass index (BMI), and history of smoking between the observation and control groups (*P* > 0.05) (see Table 1).

#### 3.1.1. Comparison of Therapeutic Effects

After treatment, the therapeutic effects were compared between the observation and control groups. There were 22 cured cases, 20 markedly effective cases, 7 effective cases, and 3 ineffective cases in the observation group, with a total effective rate of 80.77%. There were 15 cured cases, 12 markedly effective cases, 15 effective cases, and 8 ineffective cases in the control group, with a total effective rate of 56.86%. The effective rate of treatment in the observation group was remarkably higher than that in the control group (*P* < 0.05) (see Table 2).

### 3.2. Comparison of Pulmonary Function Indices before and after Treatment

**Figure 1:**
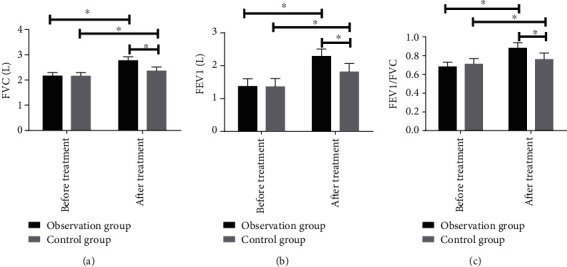
Comparison of pulmonary function indices before and after treatment: (a) the comparison of FVC before and after treatment between the observation and control groups; (b) the comparison of FEV1 before and after treatment between the observation and control groups; (c) the comparison of FEV1/FVC before and after treatment between the observation and control groups. ∗ indicates *P* < 0.05.

Before treatment, the differences were not significant in FVC, FEV1, and FEV1/FVC between the observation and control groups (*P* > 0.05). After treatment, the three indices in the two groups were remarkably improved (*P* < 0.05), but the improvement was remarkably better in the observation group (*P* < 0.05) (see Figure 1).

### 3.3. Comparison of Serum Inflammatory Cytokines before and after Treatment

**Figure 2:**
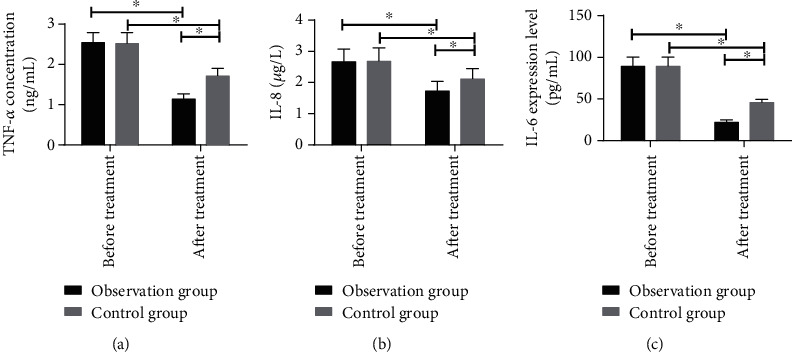
Comparison of serum inflammatory cytokines before and after treatment: (a) the comparison of TNF-*α* before and after treatment between the observation and control groups; (b) the comparison of IL-8 before and after treatment between the observation and control groups; (c) the comparison of IL-6 before and after treatment between the observation and control groups. ∗ indicates *P* < 0.05.

Before treatment, the difference was not significant in the expression of serum TNF-*α*, IL-8, and IL-6 between the observation and control groups (*P* > 0.05). After treatment, the expression in the two groups was remarkably improved (*P* < 0.05), but the improvement was remarkably better in the observation group (*P* < 0.05) (see Figure 2).

### 3.4. Comparison of Indices of Oxidative Stress before and after Treatment

**Figure 3:**
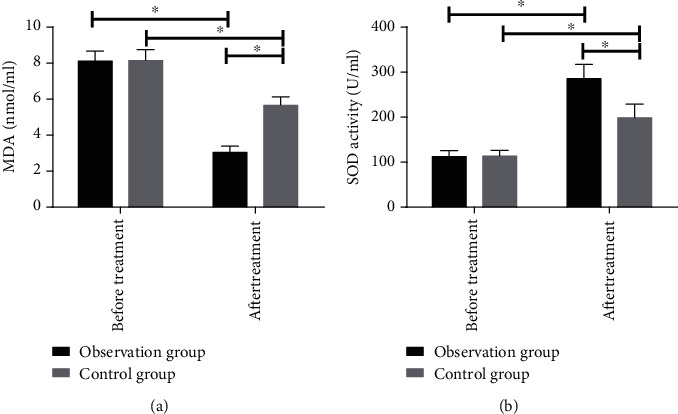
Comparison of indices of oxidative stress before and after treatment: (a) the comparison of serum MDA content between the observation and control groups; (b) the comparison of serum SOD content between the observation and control groups. ∗ indicates *P* < 0.05.

We compared contents of serum MDA and SOD before and after treatment between the observation and control groups. Before treatment, the differences were not statistically significant in the contents between the two groups (*P* > 0.05). At one week after treatment, MDA content reduced but SOD content rose in the two groups; MDA content was lower but SOD content was higher in the observation group (*P* < 0.05) (see Figure 3).

### 3.5. Comparison of Adverse Reactions

The adverse reactions of the patients during treatment were recorded and compared. In the observation group, the number of patients suffering from increased heart rate, small amount of hemoptysis, decreased blood oxygen, and decreased heart rate was 2, 2, 4, and 2, respectively, with the incidence of adverse reactions of 18.51%. In the control group, the number of patients suffering from the four adverse reactions was 2, 3, 1, and 3, respectively, with the incidence of 17.64%. The difference was not significant in the incidence of adverse reactions between the two groups (*P* > 0.05) (see Table 3).

## 4. Discussion

In recent years, with the improvement of social environment and living standards, the incidence of CI has been rising, and many CI patients usually suffer from some complications among which pulmonary infection is one of the most common ones [1, 11]. CI patients have poor respiratory and expectoration abilities, which makes their treatment more difficult when pulmonary infection occurs, so more active therapeutic methods need to be sought urgently in clinical practice [12].

In our study, the therapeutic effects of AH combined with BAL on CIPI patients were deeply analyzed. In recent years, BAL has been gradually applied to the clinical treatment of pulmonary infection, and a positive therapeutic effect of it has been achieved [13]. AH as a mucolytic can dissolve viscous phlegm and promote the secretion of pulmonary surfactant, thereby promoting sputum excretion and relieving dyspnea symptoms [14]. In this study, the combined application of AH and BAL effectively increased the effective rate of treatment and relieved the clinical symptoms of the patients. According to previous studies, the application of high-dose AH significantly improves the therapeutic effect on pulmonary infection without obvious adverse reactions [15]. BAL with a bronchoscope can directly enter the diseased region and remove inflammatory secretions in the region, thus reducing airway resistance and respiratory consumption, increasing the blood oxygenation level of the pulmonary alveolus, and improving pulmonary function [16]. In our study, AH can directly reach the diseased region through BAL, further improving the efficiency of treatment. As reported by a previous study, BAL relieves pulmonary atelectasis caused by inflammatory responses and phlegm and blood stasis obstruction in patients with pulmonary infection in a short time, thus improving pulmonary gas transfer and ventilation functions [17]. This is also consistent with our research results.

Patients with pulmonary infection suffer from more serious systemic inflammatory responses [18]. In our study, before treatment, patients in the two groups had relatively serious inflammatory responses, and the expression of TNF-*α*, IL-8, and IL-6 was remarkably higher; after treatment, the expression in the serum remarkably reduced. This indicates that the patients' inflammatory responses are remarkably reduced and that BAL with AH can further reduce the severity of inflammation in CIPI patients. Some studies have shown that the body is in an oxidative stress state when acute inflammation occurs, and oxygen consumption increases when patients suffer from pulmonary infection. At this time, the body is in a relatively hypoxic state. During this process, it produces anaerobic metabolites such as MDA, which causes further damage to the pulmonary tissue [19, 20]. SOD is a cytokine with an antioxidant effect. Its content reduces when the body has oxidative stress responses, thus weakening the body's antioxidant ability [21]. Before treatment, the oxidative stress responses in the observation and control groups were relatively strong, but they were remarkably inhibited after treatment. After treatment, compared with the control group, serum MDA content was remarkably lower but SOD content was remarkably higher in the observation group. This reveals that conventional treatment combined with AH and BAL can inhibit oxidative stress responses of CIPI patients more effectively. Finally, for further confirming the safety of BAL with AH, we compared the incidence of adverse reactions. The difference was not significant in the incidence between the observation and control groups, suggesting that BAL with AH has relatively high safety. However, in our study, the sample size is relatively small and needs further validation.

In summary, for CIPI patients, BAL with AH has a better therapeutic effect and higher safety and can control the patients' systemic inflammatory responses and oxidative stress responses, which is helpful to control and stabilize the patients' conditions, so it is worthy of further promotion in clinical practice.

## Figures and Tables

**Table 1:** General information.

Factors	Observation group (*n* = 52)	Control group (*n* = 50)	*t*/*X*^2^	*P*
Gender			0.007	0.933
Male	36 (69.23)	35 (70.00)		
Female	16 (30.77)	15 (30.00)		
Age (years)			0.197	0.657
≤56	22 (42.31)	19 (38.00)		
>56	30 (57.69)	31 (62.00)		
BMI (kg/m^2^)			0.001	0.988
≤23	28 (53.85)	27 (54.00)		
<23	24 (46.15)	23 (46.00)		
APACHEII score	22.15 ± 3.04	22.21 ± 3.08	0.099	0.921
NIHSS score	36.59 ± 3.86	36.86 ± 3.77	0.357	0.722
History of smoking			0.002	0.981
Yes	29 (55.77)	28 (56.00)		
No	23 (44.23)	22 (44.00)		
Family history of CI			0.009	0.925
Yes	15 (28.85)	14 (28.00)		
No	37 (71.15)	36 (72.00)		

**Table 2:** Comparison of therapeutic effects.

Efficacy	Observation group (*n* = 52)	Control group (*n* = 50)	*X* ^2^	*P*
Cured	22 (42.31)	15 (30.00)	—	—
Markedly effective	20 (38.46)	12 (24.00)	—	—
Effective	7 (13.46)	15 (30.00)	—	—
Ineffective	3 (5.77)	8 (16.00)	—	—
Total effective rate	42 (80.77)	27 (54.00)	8.346	0.004

**Table 3:** Comparison of adverse reactions.

Factors	Observation group (*n* = 52)	Control group (*n* = 50)	*X* ^2^	*P*
Increased heart rate	2 (3.85)	2 (4.00)	—	—
Small amount of hemoptysis	2 (3.85)	3 (6.00)	—	—
Decreased blood oxygen	4 (7.69)	1 (2.00)	—	—
Decreased heart rate	2 (3.85)	3 (6.00)	—	—
Total incidence	10 (19.23)	9 (18.00)	0.025	0.873

## Data Availability

All the raw data could be accessed by contacting the corresponding author if any qualified researcher needs.
